# Molecular Epidemiology of Clinical and Colonizing Methicillin-Resistant *Staphylococcus* Isolates in Companion Animals

**DOI:** 10.3389/fvets.2021.620491

**Published:** 2021-04-23

**Authors:** Hester Rynhoud, Brian M. Forde, Scott A. Beatson, Sam Abraham, Erika Meler, Ricardo J. Soares Magalhães, Justine S. Gibson

**Affiliations:** ^1^School of Veterinary Science, The University of Queensland, Gatton, QLD, Australia; ^2^School of Chemistry and Molecular Biosciences, The University of Queensland, Brisbane, QLD, Australia; ^3^Australian Infectious Diseases Research Centre, The University of Queensland, Brisbane, QLD, Australia; ^4^Australian Centre for Ecogenomics, The University of Queensland, Brisbane, QLD, Australia; ^5^Antimicrobial Resistance and Infectious Diseases Laboratory, College of Science, Health, Engineering and Education, Murdoch, WA, Australia; ^6^Children Health and Environment Program, UQ Child Health Research Centre, The University of Queensland, South Brisbane, QLD, Australia

**Keywords:** virulence, resistance, methicillin resistant *Staphylococcus aureus*, methicillin resistant *Staphylococcus pseudintermedius*, molecular epidemiology, colonized

## Abstract

In this study, we aimed to investigate the molecular epidemiology of methicillin-resistant *Staphylococcus aureus* (MRSA) and methicillin-resistant *Staphylococcus pseudintermedius* (MRSP) clinical and colonizing isolates of dogs and cats to profile contributing factors associated with their isolation. Nasal and rectal samples were collected from dogs and cats between 2015 and 2017 to identify colonizing isolates. Clinical isolates collected between 2003 and 2016 were retrieved from a Queensland university veterinary diagnostic laboratory. All isolates were identified using standard microbiological and molecular methods and were characterized by whole genome sequencing. Phylogenetic relationships and differences in epidemiological factors were investigated. Seventy-two MRSP isolates out of 1,460 colonizing samples and nine MRSP clinical isolates were identified. No MRSA was isolated. ST496 and ST749 were the most commonly isolated sequence types with different SCCmec types. ST496 clones spread both along the coast and more inland where ST749 was more centered in Brisbane. The resistance and virulence factors differed significantly between the two sequence types. ST496 colonizing and clinical isolates were similarly multidrug resistant. The virulence genes of ST749 colonizing and clinical isolates were similar as both contained the gene *nanB* for sialidase. There were no differences in the individual and clinical factors between predominant sequence types. High levels of antimicrobial resistance occurred in the majority of isolates, which is of potential concern to human and veterinary health. The phylogenetic clustering of isolates from this study and others previously identified in countries, particularly New Zealand, with which Australia has high volume of pet movements could suggest the importation of clones, which needs further investigation.

## Introduction

Methicillin-resistant *Staphylococcus pseudintermedius* (MRSP) and methicillin-resistant *Staphylococcus aureus* (MRSA) are concerning zoonotic opportunistic pathogens in veterinary medicine and public health. MRSP is predominately found in dogs but has been isolated from cats and humans (1, 2). In contrast, MRSA is a major human pathogen but has been identified in companion animals (3). Methicillin-resistant staphylococci (MRSs) are often multidrug resistant, which can result in infections challenging to treat. The *mecA* gene is responsible for methicillin resistance and confers reduced affinity for β-lactam antimicrobials. The gene is carried on the staphylococcal chromosomal cassette *mec* (SCC*mec*) that provides potential for transfer of resistance to other non–β-lactam antimicrobials genes between susceptible and resistant strains (4). The combination of multidrug resistance, zoonotic capabilities, and nosocomial transmission contributes to the public health concern surrounding these bacteria.

The literature has highlighted MRSA and MRSP as the most concerning multidrug-resistant staphylococci in veterinary medicine (3). There have been increasing reports of clinical and colonizing MRSP prevalence with high multidrug resistance in companion animals globally (5–7). While awareness of MRSA in companion animals has increased, this species is less commonly isolated in companion animals than MRSP (7–9). Molecular analysis has revealed that the global MRSP population is very diverse and has been identified in companion animals worldwide. Using multilocus sequence typing (MLST), studies have demonstrated between country variations in common sequence types (STs) including ST71/ST258 predominant in Europe, ST45/ST112 in Asia, and ST68 in the United States (10). All of these, with the exception of ST112, have been identified in Australian dogs and cats (6, 7). Only a few instances of MRSP colonization or infection in humans have been reported (11, 12). MRSA molecular epidemiology seems to be more clonal where major STs ST5, ST8, and ST22 have been isolated in people around the globe. Companion animals generally carry predominately human strains; ST22 and ST5 have been identified in cats and dogs from Europe, North America, and Australia, where ST8 has been identified in horses from North America and Australia (9, 13). An animal-specific strain, ST398, is more often isolated in livestock but has been identified in companion animals (13). Companion animals can act as reservoirs for MRSA and MRSP and facilitate the transmission between animals, humans, and their environments.

Both MRSA and MRSP can cause severe infections when given the opportunity. The severity of MRS infections can be directly affected by pathogen virulence factors, indirectly affected by antimicrobial resistance as infections become more difficult to treat, and by the host's immune system ability to fight the agent. MRSA and MRSP have similar virulence factors such as coagulase, cell wall components, and various toxins (14, 15). Some differ, such as the leukocidin toxins. A number of *S. aureus*–specific leukocidin toxins have been isolated, including the bicomponent Panton–Valentine leukocidin, which is generally identified in the more virulent strains (16). The leukocidin toxin identified in MRSP does not seem to be highly associated with disease and is usually isolated in all *S. pseudintermedius* isolates (17). Both MRSA and MRSP display resistance to β-lactam and many non–β-lactam antimicrobial classes, such as fluoroquinolones, aminoglycosides, tetracyclines, sulfonamides, macrolides, and lincosamides (18–22). However, MRSP isolates from companion animals have been reported to be more multidrug-resistant than MRSA (23). A recent study has revealed that many clinical MRSP multidrug-resistant clones have emerged through multiple independent, horizontal acquisition of resistance determinants and frequent genetic exchange that disseminate DNA to the broader population (24). It is crucial to have close epidemiological and microbiological surveillance on MRSP in companion animals in place to determine their burden to companion animals and infection control in veterinary hospitals and whether they have potential to be a threat to public health.

While the molecular epidemiology of MRSA and MRSP in small animals has been explored extensively globally, very few have combined the assessment of both clinical and carriage isolates (17, 25–27). This study analyzes the relationship between the molecular population structure, including virulence and resistance genes of clinical and colonizing isolates and any associated epidemiological risk factors. The identification of epidemiological (i.e., demographic, clinical, and genetic) characteristics of major clones associated with colonization can assist with surveillance efforts toward the potential for emergence of these strains as clinical pathogens to companion animals and humans.

This research aimed to investigate the molecular epidemiological characteristics of MRSA and MRSP isolated in a population of dogs, cats, and horses that presented to veterinary clinics and shelters in South East Queensland (SE QLD), Australia. Specific objectives included a comparison between colonizing and clinical isolates with respect to their demographic and clinical characteristics, resistance and virulence genes, and where they genetically fit in the global phylogeography of *S. pseudintermedius*.

## Materials and Methods

### Sample Population and Attribute Data

This research was approved by the Animal Ethics Unit from the University of Queensland (the University of Queensland Animal Ethics SVS/487/15/KIBBLE). To obtain MRS colonizing isolates, nasal and rectal samples were collected between November 2015 and December 2017, with owner's written consent, from dogs and cats that presented to five veterinary clinics and were housed in three shelter facilities. Animals were sampled at clinics if they had not been hospitalized for more than 24 h to minimize the likelihood of isolating hospital-associated strains. Animal demographic and clinical history data [date of sampling (day/month/year), signalment, household geographical location, consultation types, and previous antimicrobial and corticosteroid use] up to 12 months prior to sampling were extracted from each animal's medical record.

Data from the University of Queensland's veterinary diagnostic laboratory collection was searched between 2003 and 2016 to identify clinical samples of MRSs. Demographic or clinical history data were only available for some cases. The populations from which colonizing and clinical isolates were obtained were similar as both were from SE QLD, and majority were from dogs.

### Sample Processing

After sample collection, the transport media swabs were stored at 4°C and transported to the laboratory for processing within 5 days from collection. Swabs were incubated in Mueller Hinton Broth containing 6.5% NaCl (wt/vol) (Thermo Fisher Scientific, Thebarton, South Australia, Australia) aerobically overnight. Clinical samples that were previously identified as MRSA or MRSP were retrieved from the −80°C freezer and incubated in the Mueller–Hinton broth aerobically. *Staphylococcus* isolation and identification were achieved using routine microbiological tests including selective MRSA 2 Brilliance™ agar (Thermo Fisher Scientific), Gram staining, and catalase and coagulase testing. Clinical isolates also underwent Staphytect Plus (Oxoid) and other biochemical tests (Voges-Proskauer and Ortho-nitrophenyl-b-d-galactopyranoside).

Susceptibility testing was performed on 13 antimicrobials [amikacin (AMK), clindamycin (CLI), chloramphenicol (CHL), enrofloxacin (ENR), erythromycin (ERY), gentamicin (GEN), imipenem (IPM), mupirocin (MUP), penicillin (PEN), tetracycline (TET), trimethoprim/sulfamethoxazole (SXT), vancomycin (VAN), cefoxitin (FOX), and oxacillin (OXA)] using the disc diffusion method as per the Clinical and Laboratory Standards Institute guidelines (28, 29). If an isolate was not susceptible to at least one agent in at least three antimicrobial categories, then it was considered multidrug-resistant (30).

### Detection of mecA and Molecular Characterization

OXA- and FOX-resistant isolates were subjected to a multiplex polymerase chain reaction to identify the *mec*A gene and to distinguish between MRSA and MRSP (31, 32). All *mec*A-positive isolates were submitted for Illumina sequencing at Murdoch University in Australia. Paired-end sequencing libraries were prepared using the Illumina Nextera XT Library Preparation kit. Whole genome sequencing (WGS) was performed on the Nextseq 500 platform (150-bp paired-end). Raw Illumina sequencing data was quality filtered (trimmomatic version 0.36) (33) to remove Illumina adaptor sequences, low-quality bases (phred quality <10), and reads shorter than 50 bp. Quality filtered Illumina reads were assembled *de novo* using SPAdes v3.12.0 (34) with default parameters.

Species identification was confirmed with WGS data. *In silico* sequence typing (ST) was performed using SRST2 (35) and the *S. pseudintermedius* typing scheme available from PubMLST^1^. Novel STs were submitted to the MLST database curator (vincent.perreten@vetsuisse.vbi.unibe.ch). SCC*mec* typing was performed using *de novo* assembled contig sequences for each isolate and SCCmecFinder (*https://cge.cbs.dtu.dk/services/SCCmecFinder*) (37). Resistance gene profiling was performed by screening Illumina sequence reads against the NCBI Bacterial Antimicrobial Resistance Reference Gene Database (38) using SRST2 version 0.2.0 (35). All isolates were screened for mutations in the topoisomerase II (*gyrA*) and IV genes (*grlA*) associated with decreased susceptibility to fluoroquinolones. Virulence gene profiling was performed by screening Illumina sequence reads against a customized Virulence Factor Database (39), modified to include *S. pseudintermedius* virulence factors, using SRST2. Spa gene homologs (*spsP* and *spsQ*) were manually identified using Artemis Comparison Tool (40) to compare assembled contigs with the *spa* locus (between SPSE_0038 and *spsL*) from the *S. pseudintermedius* strain ED99 reference genome (accession no. CP002478).

A phylogenetic tree was constructed using a core SNP alignment following the removal of recombinant regions. Draft genomes were aligned using Parsnp v1.237 (41), one of which was randomly selected to act as a reference, generating a core genome alignment. Recombinant regions were predicted and removed using Gubbins v2.1.038 (42). The phylogenetic tree was generated using RAxML v8.2.939 (43) using a general-time reversible nucleotide substitution model with a GAMMA correction for site variation. The phylogenetic tree was rooted using a *Staphylococcus delphini* genome (www.ncbi.nlm.nih.gov; accession no. NZ_LR134263) and visualized as a cladogram using FigTree v1.4.2 (44) and Evolview v2 (45). A second global tree was created using both the MRSP isolates from this study and all other *S. pseudintermedius* available in GenBank (accessed February 3, 2020).

### Statistical Analyses

Fisher exact tests were used (due to small sample sizes) to quantify pairwise differences in the proportions of common MRSP clones with particular animal [i.e., sex, age, neuter status, species (dog or cat), sampling location (clinics A, B, C, and an animal shelter), antimicrobial and corticosteroid use in the year prior to sampling, consultation type] and genotypic attribute data (i.e., resistance and virulence genes) (Supplementary Table 1). The resistance genes were classified into three groups according to their resistance to first-line, second-line, third-line, and important human antimicrobials (Supplementary Table 2) (46). Virulence genes were grouped into six categories based on the similarity of virulence gene profiles (Supplementary Table 2). MRSP clones were categorized according to their shared multilocus STs and SCC*mec* types.

To quantify pairwise differences between the common MRSP clones and the clinical isolates in regard to their resistance and virulence genes, we used the Fisher exact test with an adjusted significance *p*-value of 0.025 (to account for a total of two comparisons) (47).

### Mapping Local and Global Isolates

We generated a map showing the geographical distribution of common MRSP clones in the study area and a map of the worldwide distribution of shared STs of genetically related *S. pseudintermedius* (both methicillin-resistant and susceptible) using ArcMap version 10.6.1. Study isolates and isolates from the worldwide *S. pseudintermedius* GenBank database were grouped together by their clusters in the phylogenetic tree. Not all isolates had STs available, and so if they clustered with a known ST in the phylogenetic tree, they were grouped together.

## Results

### Prevalence of MRS in Our Study Sample

A total of 1,460 samples were collected from 678 participating animals (Supplementary Table 3). No MRSA was isolated in cats or dogs. There were 51 MRSP colonizing isolates in 34 of 409 dogs (8%) from the population presenting to the clinics. Sixteen of the isolates were from 11/255 dogs (4%) sampled at clinic A; 24 isolates were from 14/102 dogs (14%) from clinic B, and 11 isolates were from 9/30 dogs (30%) sampled at clinic C were positive for MRSP. No MRS was isolated from animals presenting to other clinics. Twenty-one MRSP isolates were from shelter animals. Nine isolates were isolated in 7/61 dogs (11%) and 12 isolates from 9/127 cats (7%). All the positive animals were from the same shelter. A total of 95 clinical staphylococcal isolates were identified, and nine of these were identified as MRSP. Eight of these isolates were sampled from eight dogs at veterinary clinics: one in 2007, another in 2009, three in 2013, and another three in 2016. The ninth isolate was isolated from a dog in a shelter in 2017. In total, there were 81 MRSP isolates.

### Antimicrobial Susceptibility

Majority of the MRSP isolates were phenotypically resistant to TET (colonizing: 72%, clinical: 67%), SXT (colonizing: 68%, clinical: 78%), CHL (colonizing: 63%, clinical: 78%), ENR (colonizing: 69%, clinical: 56%), ERY (colonizing: 70%, clinical: 100%), and CLI (colonizing: 55%, clinical: 100%) (Table 1). The most commonly detected profile in colonizing (40%) and clinical (44%) isolates included resistance to TET, SXT, CHL, ENR, ERY, and CLI, with the addition of GEN in clinical isolates.

**Table 1:** The percentage of all methicillin-resistant *Staphylococcus pseudintermedius* (MRSP) isolates from this study that displayed phenotypic resistance to 11 antimicrobials.

**Groups**	**TET**	**SXT**	**CHL**	**ENR**	**ERY**	**CLI**	**GEN**	**AMK**	**VAN**	**IPM**	**MUP**	**MDR**
Colonizing isolates	72	68	63	69	70	55	4	0	0	0	1	73
Clinical isolates	67	78	78	56	100	100	67	0	0	0	0	100

*All isolates were resistant to penicillin (10 units) and oxacillin. Oxacillin (OXA 1 μg) antimicrobial susceptibility was determined for all suspected S. pseudintermedius isolates (28, 48). All isolates resistant to oxacillin were assumed resistant to all currently available β-lactam antimicrobial agents, with the exception of the newer cephalosporins with anti MRSA activity, in accordance with the CLSI guidelines. Resistance included isolates that were intermediately resistant. TET, tetracycline (30 μg); SXT, trimethoprim-sulfamethoxazole (1.25/23.75 μg); CHL, chloramphenicol (30 μg); ENR, enrofloxacin (5 μg); ERY, erythromycin (15 μg); CLI, clindamycin (2 μg); GEN, gentamicin (10 μg); AMK, amikacin (30 μg); VAN, vancomycin (30 μg); IPM, imipenem (10 μg); MUP, mupirocin (200 μg); MDR, multidrug resistant*.

### Molecular Characteristics of MRSP

Eleven different STs were identified, two of which were novel (ST1399 and ST1400) (Table 2). The most common ST in the colonizing isolates included ST496 (*n* = 43) and ST749 (*n* = 13). All the ST496 isolates carried a subtype of SCC*mec* type V (5C2&5), and the ST749 isolates carried SCC*mec* type IVg (2B). The majority of clinical isolates were ST316-III (3A) (*n* = 5). Ninety-seven percent to 100% of isolates harbored genes conferring resistance to β-lactams (*blaZ, mecA*); no isolates carried the *mec*C gene. Ninety-seven percent to 100% of isolates contained the following genes and displayed the corresponding phenotypic resistance; *cat-pC221, catA7* (CHL), *ermB, ermC* (macrolides and lincosamides), *tetM* (TETs), *dfrG* (sulphonamides), and mutations in their *grlA* and *gyrA* genes (fluoroquinolones). Only 25% of isolates carrying the resistance genes for aminoglycosides (*aac6-aph2, ant6-Ia, aph3-III*, and *aadD)* conferred resistance to GEN, and no isolates were phenotypically resistant to AMK.

**Table 2:** The molecular characteristics of both clinical and colonizing methicillin-resistant *Staphylococcus pseudintermedius*.

**MLST**	**Colonizing or clinical**	**SCCmec type**	**Resistance genes^a^**	**Phenotypic resistance (including intermediate resistance)^b^**
71 (n = 6)	Colonizing (n = 3) Clinical (n = 1)	III(3A) (n = 4)	*aac6-aph2, aadD* (n = 1), *ant6-Ia, aph3-III, sat4A, blaZ, mecA, ermB, ermC* (n = 1), *cat-pC221* (n = 2), *dfrG, grlA, gyrA*	SXT, CHL (n = 2), ENR, ERY, CLI, GEN (n = 3), MUP (n = 1), OXA
	Colonizing	V(5C2) (n = 1)	*aadD, ant6-Ia, aph3-III, sat4A, blaZ, mecA, ermB, ermC, cat-pC221, dfrG, grlA, gyrA*	SXT, ENR, ERY, CLI, GEN, OXA
	Colonizing	V(5C2&5)|subtype Vc(5C2&5) (n = 1)	*aac6-aph2, ant6-Ia, aph3-III, sat4A, blaZ, mecA, ermB, dfrG, grlA, gyrA*	SXT, ENR, ERY, CLI, GEN, OXA
84 (n = 3)	Colonizing	Va(5C2)	*ant6-Ia, aph3-III, sat4A, blaZ, mecA, ermB, tetM* (n = 2)	TET, ERY, CLI, OXA
153 (n = 1)	Colonizing	Va(5C2)	*ant6-Ia, aph3-III, sat4A, blaZ, mecA, ermB, tetM*	TET, ERY, CLI, OXA
258 (n = 1)	Colonizing	IVg(2B)	*ant6-Ia, aph3-III, sat4A, blaZ, mecA, tetM, dfrG*	TET, SXT, OXA
276 (n = 1)	Colonizing	Va(5C2)	*blaZ, mecA*	OXA
283 (n = 2)	Clinical	Va(5C2)	*ant6-Ia, aph3-III, sat4A, blaZ, mecA, ermB, grlA* (n = 1)	ERY, CLI, OXA
316 (n = n = 8)	Colonizing	II(2A) (n = 1)	*aac6-aph2, ant6-Ia, aph3-III, sat4A, blaZ, mecA, ermB, cat-pC221, tetM, dfrG, gyrA*	TET, SXT, CHL, ENR, ERY, CLI, GEN, OXA
	Clinical	III(3A) (n = 5)	*aac6-aph2, ant6-Ia, aph3-III, sat4A, blaZ, mecA, ermB, cat-pC221, tetM, dfrG, grlA* (n = 1), *gyrA*	TET, SXT, CHL, ENR, ERY, CLI, GEN, OXA
	Colonizing	Va(5C2) (n = 2)	*aac6-aph2, ant6-Ia, aph3-III, sat4A, blaZ, mecA, ermB, cat-pC221, tetM*, dfrG, gyrA	TET, SXT, CHL, ENR, ERY, CLI, GEN, OXA
496 (n = 43)	Colonizing (n = 42) Clinical (n = 1)	V(5C2&5) subtype	*aac6-aph2* (n = 36), *ant6-Ia, aph3-III, sat4A, blaZ, mecA, ermB, cat-pC221* (n = 38), *tetM, dfrG, grlA, gyrA*	TET (n = 42), SXT (n = 42), CHL (n = 42), ENR (n = 42), ERY (n = 42), CLI (n = 37), GEN (n = 3), OXA
749 (n = 13)	Colonizing	IVg(2B)	*blaZ, mecA*	OXA
1,399 (n = 2)	Colonizing	IVg(2B)	*blaZ, mecA, tetM*	TET, OXA
1,400 (n = 1)	Colonizing	IVg(2B)	*blaZ, mecA*	OXA

a *grlA and gyrA refer to a mutation in these two genes that confer resistance to fluoroquinolones*.

b *All isolates were resistant to penicillin. All MRSP isolates were resistant to oxacillin. Resistance included isolates that were intermediately resistant. TET, tetracycline; SXT, trimethoprim-sulfamethoxazole; CHL, chloramphenicol; ENR, enrofloxacin; ERY, erythromycin; CLI, clindamycin; GEN, gentamicin; AMK, amikacin; VAN, vancomycin; IPM, imipenem; MUP, mupirocin*.

The most common virulence genes identified included toxins [*lukF-P, hlgB* (gamma hemolysin component B)], exfoliative toxin [*siet* (*Staphylococcus intermedius* gene for exfoliative toxin), *speta* (exfoliative toxin A)], accessory gene regulators [s*rrA* (staph respiratory response protein), *sarA* (staphylococcal accessory regulator A), *sarR* (transcriptional regulator), *sarZ* (transcriptional regulator)], cell wall anchored proteins [*ebpS* (elastin-binding protein), *spsC, spsE, spsH, spsK, spsA, spsB, spsG, spsI, spsM, spsN, spsQ*, and *spsR* (*S. pseudintermedius* surface proteins)], exoenzymes [*nanB* (putative sialidase toxin), *coa* (staphylocoagulase), *hrtA* (heme efflux system ATPase HrtA), *lip* (triacylglycerol lipase)], *clpX* [ATP-dependent protease ATP-binding subunit ClpX], and a protease *clpP*. *nanB* was only present in four STs (ST84, ST153, ST316, and ST749); *spsI* was only present in three STs (ST316, ST749, and ST1399); and *expB* (exfoliative toxin B) was identified in a single ST749 isolate. The *spa* gene ortholog *spsQ* was identified in ST71, ST283, ST316, ST496, and ST1399 isolates. In ST496, *spsQ* was deleted in 658bUQ and 344aUQ. The homologous *spsP* gene (encoded immediately upstream of *spsQ*) was present in ST316 and ST1399 isolates, but carried a frameshift in ST71 isolates, and a 5′ deletion in ST283 isolates. In ST496 isolates, *spsP* was truncated at the 5′ end in the majority of cases or deleted entirely in the ST496 subclade that includes 724aUQ and 307bUQ2. MRSP that lacked the *spa* locus exhibited either a 4.5-kb deletion between SPSE_0039 and SPSE_0044 (ST84 and ST276) or a 7.8-kb deletion between SPSE_0039 and *spsL* (ST749 and ST1400).

### Phylogeny of MRSP and Associated Metadata

**Figure 1:**
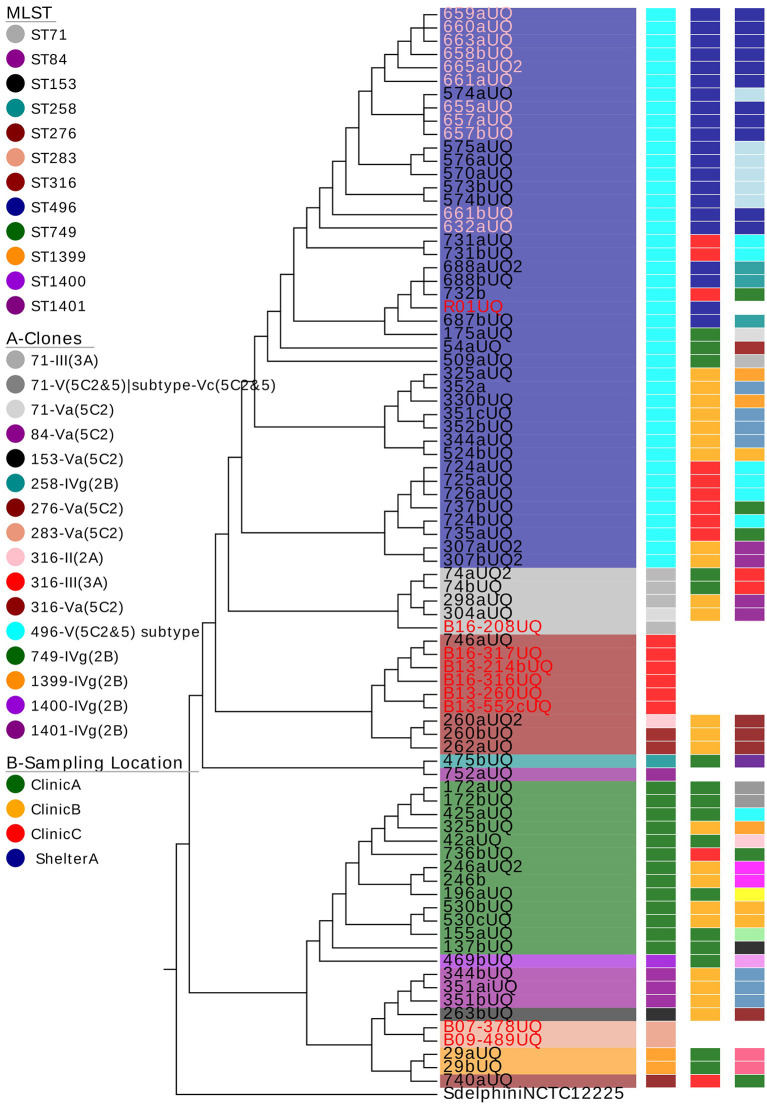
The phylogenetic tree of all methicillin-resistant *Staphylococcus pseudintermedius* (MRSP) isolates and associated metadata. The colors behind the isolate names represent the multilocus sequence types (MLST). The isolates highlighted in pink are MRSP isolated from cats. The rest of the isolates are isolated from dogs. The isolates highlighted in red are clinical isolates. The column labeled A = MRSP clones with the same MLST and SCC*mec* types; B = the sampling location, C = the date of sampling. The colors used in column C indicate a different sampling date (i.e., same day = same color). Samples 425 and 172 were isolated from the same dog.

From the annotated phylogenetic tree (Figure 1), it is evident that there was more diversity in MLST-SCC*mec* clonal types isolated from clinic A (seven clones) and clinic B (nine clones), followed by clinic C (four clones) and shelter A (three clones). The isolates at shelter A all belonged to ST496. There was no pattern identified in terms of sampling dates.

**Figure 2:**
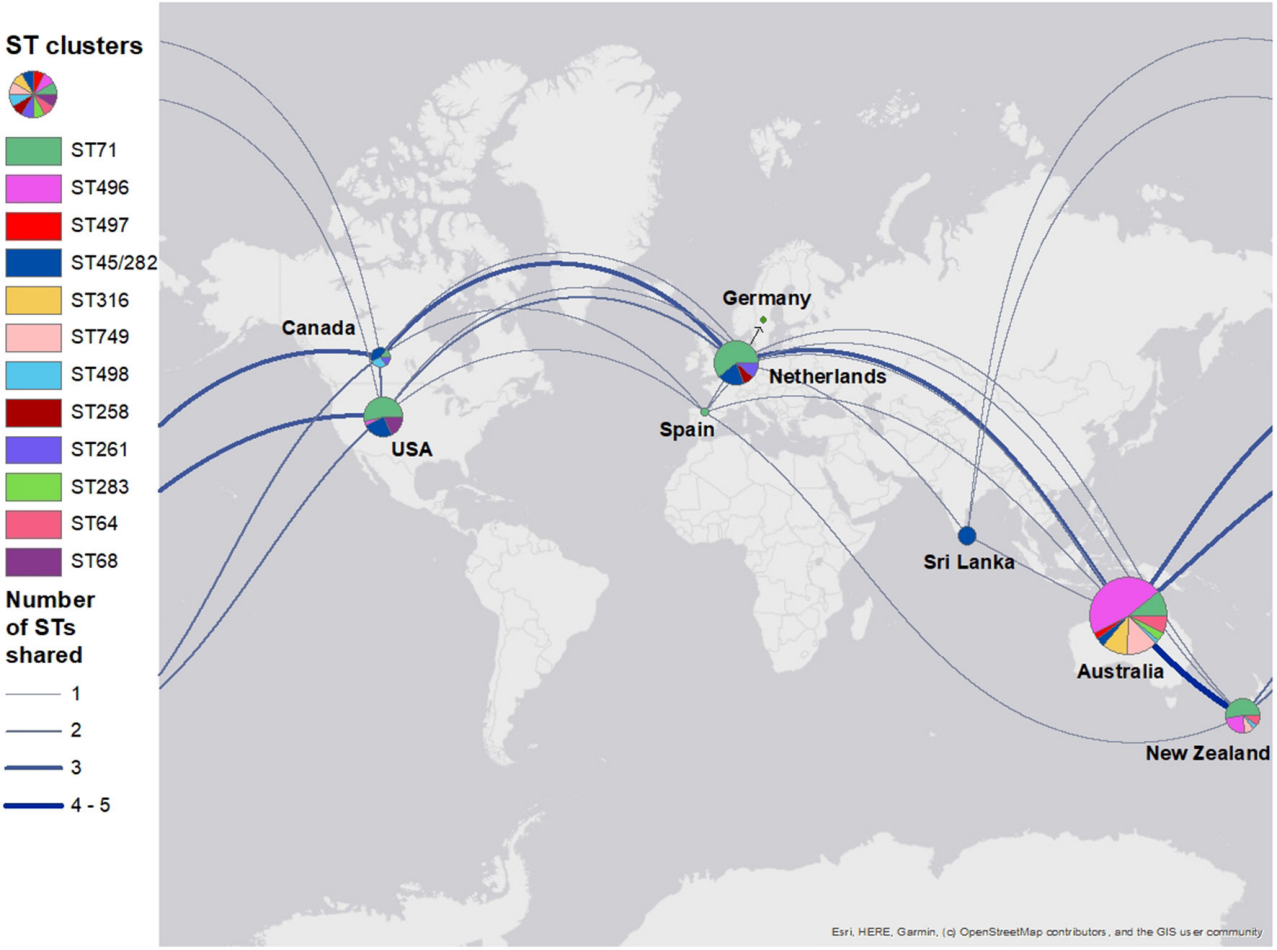
A world map displaying shared methicillin-resistant *Staphylococcus pseudintermedius* sequence types among countries. Isolates were grouped into sequence type clusters according to how they clustered in a phylogenetic tree. The pie chart for Germany was covered by the Netherlands pie chart and so has been pointed out with an arrow. Pie chart sizes reflect the number of isolates from each country (i.e., a bigger chart reflects a larger number of isolates).

In total, the global tree had 35 different STs and 133 isolates with unidentified STs (Supplementary Figure 1). The isolates were collected from 20 different countries (United States, the Netherlands, Australia, New Zealand, Canada, Sri Lanka, Botswana, Denmark, South Korea, Japan, United Kingdom, Argentina, Belgium, Czech Republic, Germany, Grenada, Hong Kong, Ireland, Israel, Spain, and Sweden). There were isolates from 202 dogs, 11 humans, 7 environmental, 5 horses, and 3 cats, and one was of unknown origin. These included 162 clinical samples and 46 colonizing samples; clinical or colonization status was not specified for 14 isolates. The majority of the isolates from this study, particularly ST496, ST749, and ST316, clustered closely with other clinical and colonizing isolates from Australian and New Zealand dogs. The global map of different STs indicated that Australia shared STs from different countries (Figure 2). Australia and New Zealand shared more STs than other countries (ST496, ST71, ST749, ST498, and ST64). This is followed by Canada (ST71, ST45/282, and ST498), the Netherlands (ST71, ST45/282, and ST497), and the United States (ST71, ST64, and ST45/282).

### Epidemiological and Genomic Differences Between Common MRSP Colonizing Clones

**Figure 3:**
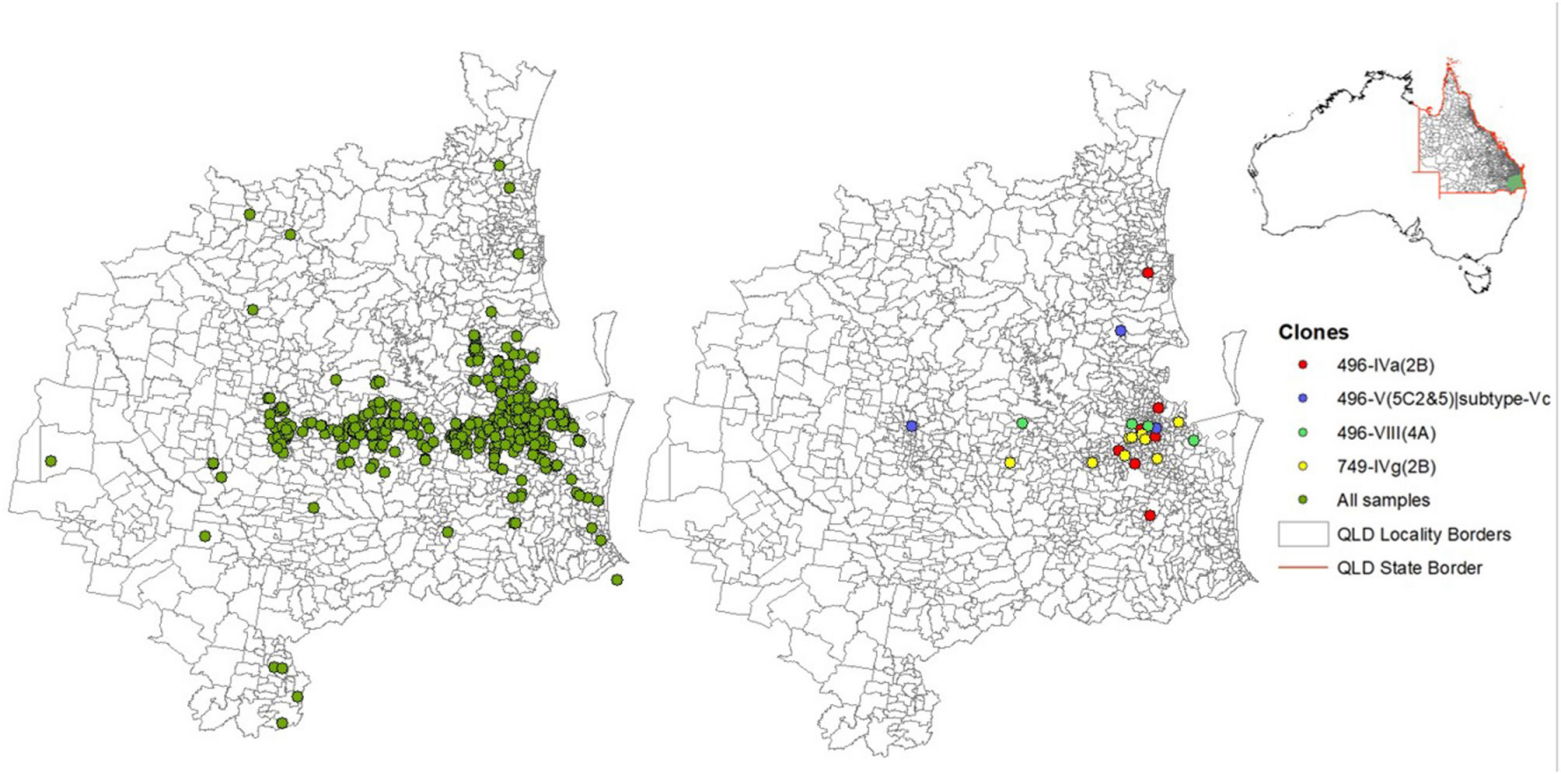
A map showing the distribution of sampled animals and common methicillin-resistant *Staphylococcus pseudintermedius* clones. The two maps with sampled points correspond to the green area highlighted in the map of Australia, within the Queensland (QLD) border.

The majority of MRSP clones were recovered from dogs compared to cats, and ST749 clone was exclusively isolated from dogs (Supplementary Table 1). The geographic distribution of the two common clones in SE QLD indicated that ST496 isolates were dispersed throughout the landscape of SE QLD, whereas ST749 isolates were mainly aggregated around the Brisbane metropolitan area (Figure 3).

Sampling location was significantly different between ST496 and ST749 isolates (*p* = 0.001) (Table 3). The resistance and virulence genes were significantly different between ST749 and ST496 isolates (*p* ≤ 0.001).

**Table 3:** Epidemiological differences between most common colonizing methicillin-resistant *Staphylococcus pseudintermedius* clones and between these and nine clinical isolates using Fisher exact test; results are expressed as *p*-values for each pairwise comparison.

**Clone combinations**	**Sex^1^**	**Species^2^**	**Age^3^**	**Neuter status^4^**	**Sampling location^5^**	**Antimicrobial use^6^**	**Corticoid use^7^**	**Consult type^8^**	**Resistance^9^**	**Virulence^10^**
496 vs. 749	0.063	0.173	0.255	0.710	0.001^a^	0.219	1	0.007	<0.001^a^	<0.001^a^
496 vs. clinical									0.173	<0.001^b^
749 vs. clinical									<0.001^b^	0.083

a *p < 0.005 and*

b *p < 0.025 are significant*.

1 *= Female or male*;

2 *= dog or cat*;

3 *= age category (≤1, 1–4, 4–7, 7–, and ≥10 years)*;

4 *= neutered or entire*;

5 *= shelter, clinic A, clinic B, or clinic C*;

6 *= whether antimicrobials were used within year prior to sampling*;

7 *= whether glucocorticoids were used within year prior to sampling*;

8 *= general practice, internal medicine, dermatology, surgery or shelter animals*;

9 *= six categories according to resistance genes*;

10 *= six categories according to virulence genes*.

Comparison of ST membership and colonization of antimicrobial resistance genes indicated that between ST749 isolates and the nine clinical isolates the resistance gene profile was significantly different (*p* < 0.001), but virulence genes were not (Table 3). Further, our results also indicated that the virulence genes of ST496 were significantly different from that of clinical isolates (*p* < 0.001). There were not enough data to do meaningful comparisons between clinical isolates and epidemiological factors.

## Discussion

This study demonstrated a similar prevalence of MRSP colonization in dogs sampled at the five clinics (8%) and dogs sampled in the three shelter environments (11%). No cats sampled in clinics were MRS positive, but 7% of shelter cats sampled were positive for MRSP. These colonization rates fall within the range reported in the broader literature, 3–34% in dogs and 4–19% in cats (7, 49–54). No MRSA was isolated from companion animals in this study; this result is expected as colonization has been reported to range between 0.5 and 9% in dogs and 0 and 4% in cats (55–59).

The MRSP isolates in our study population belonged to 11 different STs, two of which were novel (ST1399 and ST1400). The most common colonizing ST included ST496 and ST749, both of which have been described recently in Australia (6, 7). The distribution of ST496 clones in our study was widespread compared to ST749, which was more centered around Brisbane. ST496 was first reported in Australia from isolates sampled in 2013 and was also the predominant clone in a dog population from Sydney, New South Wales (6, 7). This differs to the rest of the world where ST71/ST258 is often reported in Europe, ST45/ST112 in Asia, and ST68 in the United States (10). ST71 was reported in six of our colonizing isolates, and only one was ST258; none of the other major STs were isolated. In contrast, a previous Australian study reported that the most common ST identified in their MRSP clinical isolates belonged to ST71; however, the majority of them came from Victoria (6). The same study also reported a few isolates with ST45 and a single ST258. This could suggest the expansion of a local Australian clone and possible clustering of different clones in the different states as described by Worthing et al. (6). ST496 has also been described in New Zealand and France, and ST749 has been described in New Zealand (60, 61). ST496 could be an emerging major ST that could replace others, as ST258 and ST551 have replaced ST71 in France and Poland (60, 62).

The diverse global population structures of *S. pseudintermedius* are evident in the world map comparing shared STs. ST71 is still present and dominant in a number of countries, especially in Europe. Although ST45 was previously described as a predominately Asian clone, it is now present in the United States, Canada, the Netherlands, Sri Lanka, and Australia. ST749 is unique to Australia and New Zealand. ST496 also seems to be unique to these two countries, although we know it has also been reported in France (60). We suggest that pet migration between SE QLD, Australia, and other countries, particularly New Zealand, is likely to play an important role in the clone profile observed in this study. This hypothesis is supported by data from the Australian Government Department of Agriculture, Water and Environment as the majority of cat and dog imports and exports to and from Australia were linked to New Zealand (63, 64). Between 2015 and 2019, 25–30% of cats and dogs exported from Australia were to New Zealand, and between 2016 and 2019, 34–36% of dogs and cats imported into Australia were from New Zealand. The United Kingdom and the United States were next with imports and exports ranging between 11 and 16% for both countries. Future research could consider investigation of potential links between pet movements into Australia from different countries and the molecular epidemiology of specific MRSP clones and the biosecurity implications of this.

ST496 was first described in Australian clinical MRSP isolates from companion animals in 2018 (6). It was later identified in clinical isolates from dogs in France and New Zealand (60, 61). Although these studies did not report SCC*mec* types, they did report multidrug resistance, which is consistent with our data. A Sydney study that focused on MRS colonization in companion animals identified ST496 clones with SCC*mec* types Vt, which could suggest the presence of geographically localized clones (7). SCC*mec* carried by the ST496 isolates in this study was also similar to SCC*mec* type Vt (MRSA strain PM1 NCBI assembly accession no. GCA_000308895.1), which is a representative of type V (5C2&5) (65). Examination of the contigs encoding the SCC*mec* element within ST496 isolates revealed that the J1 junction region encodes a subtype IIIA CRISPR locus and therefore differs from other type V (5C2&5) SCC*mec* in the SCCmecFinder database. This type V (5C2&5) variant has been previously reported in *Staphylococcus capitis* CR01 as part of a composite element (66) and is found in a number of publicly available staphylococcal genomes including *S. pseudintermedius* strains AI14 (CP031604) and AP20 (CP031561). Indeed, there seemed to be geographical-related differences between the ST496 and ST749 isolates. ST496 was isolated in significantly different locations to ST749. All ST749 samples were isolated on seven different days from clinic A, three different days from clinic B, and on a single day from clinic C. All of the shelter animal isolates were an ST496; however, ST496 clones were also present in dogs outside of the shelter. ST496 isolates from the shelter animals were isolated on three separate days. This suggests possible community transmission events, although we do not have enough data to determine whether the animals were in contact with another or whether the environment was contaminated. MRSP has been isolated from environmental samples before; however, a recent study could not identify any in Australian veterinary hospitals (7, 19). It is also possible that veterinary personnel could be acting as carriers that transmit the bacteria between animals that they handle, or pet owners who interact with other dogs in the community such as dog parks. Future work should aim to investigate prevalence of MRS in the environment and people interacting with the animals in order to analyze transmission dynamics of MRSP. The distribution of the ST496 clones was widespread both along the coast and more inland where ST749 was more centered in Brisbane.

Our results demonstrate that ST496 isolates were very similar in their multiresistance pattern with resistance to aminoglycosides (*ac6-aph2, ant6-Ia, aph3-III*), streptothricin (*sat4A*), β-lactams (*blaZ, mecA*), macrolides (*ermB*), CHL (*cat-pC221*), TET (*tetM*), and trimethoprim (*dfrG*). The presence of these genes was well-correlated with phenotypic sensitivity results; however, very few ST496 isolates that carried *ac6-aph2* conferring resistance to GEN were phenotypically resistant to GEN. These genes were reported in the other studies on ST496 strains, suggesting that their multidrug resistance could have contributed to their widespread dissemination compared to other STs (6, 60, 61). However, our study shows that the resistant genes of ST749 isolates were significantly different to that of ST496 isolates. They were much less resistant and only harbored β-lactam resistance genes (*blaZ, mecA*), which may account for the fact that these clones are often found in healthy dogs (7). The ST749 isolates were phylogenetically clustered with two novel STs (ST1399 and ST1400), which had similar resistomes and the same SCC*mec* types, and were isolated from the same sampling location (clinic A). This could suggest possible transfer of the SCC*mec* element between MRSP isolates. It was interesting to see that some animals harbored different clones simultaneously. Further longitudinal investigations are needed to ascertain whether one clone predisposes an animal to have another.

The majority of clinical and colonizing isolates belonging to the predominant clones carried a similar range of virulence genes. These included genes encoding for cytotoxic toxins (*lukF-P, hlgB*), exfoliative toxins (*siet, speta*), accessory gene regulators (s*rrA, sarA, sarR*, and *sarZ*), cell wall anchored proteins (*ebpS, spsC, spsE, spsH, spsK, spsA, spsB, spsG, spsI, spsM, spsN, spsP, spsQ*, and *spsR*), and exoenzymes (*nanB, coa, hrtA, lip, clpX*, and *clpP*) (60, 67–70). There were statistically significant differences in virulence genes between ST496 and ST749 isolates, and between ST496 colonizing isolates and the nine clinical isolates. The variances in virulence genes between these three groups were subtle, with the obvious difference being the presence of *nanB* in ST749 isolates, which was also isolated in five of the nine clinical samples. The *nanB* gene for sialidase may contribute to host colonization by providing a carbon source for growth, contributing to biofilm formation, or by enhancing adherence by exposing receptors on the host cell (69). Sialidase enzymes help regulate sialic acid on cell surfaces, which are important determinants of eukaryotic cell–cell interactions (71). Perhaps this virulence gene has played a role in the success of this ST. The two exfoliative toxins identified included *S. pseudintermedius* exfoliative toxin (SIET) and SPETA. There is conflicting evidence regarding the role of SIET in canine pyoderma as a previous study that injected SIET into dogs showed an association with clinical signs seen in canine pyoderma (erythema, exfoliation, and crusting) where another reported a lack of association (34). SPETA was identified as an exfoliative toxin due to its high amino acid similarity to existing exfoliative toxins SHETA from *Staphylococcus hyicus* and ETA from *S. aureus*; however, its functionality as an exfoliative toxin is yet to be determined (69, 72). Both of these toxins are often reported in *S. pseudintermedius* isolates, including more than 89% of isolates in this study, so future studies investigating their roles in disease processes are warranted. The staphylococcal protein A (spa) homolog, *spsQ*, was present in the majority of isolates in this study. The spa region in *S. pseudintermedius* encodes another *spa* homolog, *spsP*, tandemly arranged upstream of *spsQ*. In most isolates, *spsP* was deleted or contained a frameshift mutation, with some STs lacking both *spa* genes due to deletions. These results are consistent with a recent report highlighting the *spa* locus as a hotspot for deletion and recombination (73). Protein A has been associated with diseased dogs in previous studies (74). The virulence genes were similar in most isolates and bar the presence of *nanB, expB*, and *spsQ*, and so it is not clear whether some STs were more virulent than others.

The results of this study need to be interpreted in light of its limitations, which indicate important directions for future work in a number of areas. While our analyses revealed differences between clinical and colonizing isolates in terms of their resistance and virulence, our clinical isolate data were not sufficient to make meaningful comparisons between the two groups. More research is warranted in this area as it can be beneficial to determine whether certain factors increase the probability of colonizing isolates causing infections. Our analysis comparing *S. pseudintermedius* isolates from different countries is limited to the availability of isolates in the GenBank database. Although some of the STs identified in this study have also been isolated in other countries, such as ST496 in France (60), the lack of publicly available WGS data prevented the inclusion of such isolates in this study.

## Conclusions

Our study demonstrates that a sample population of healthy pets from SE QLD is primarily carriers of ST496 and ST749 MRSP clones, and these STs differ significantly in their resistance and virulence patterns. Their success could be partly explained by our findings that ST496 was found to harbor high levels of resistance and that ST749 had similar virulence genes to clinical MRSP isolates such as the presence of the *nanB* gene, which might help with colonization. Because of the high levels of resistance reported in the majority of clinical and colonizing isolates, veterinarians treating MRSP infections will most likely come across MDR isolates, which could hinder treatment. ST496 is widely distributed across SE QLD, and both strain types, as well as others, have similarities to clones previously identified in New Zealand and other countries with which Australia has high volume of pet movements. Importation of highly resistant strains could lead to infections that are difficult to treat. The biosecurity implications of these findings need further investigation.

## Data Availability Statement

The datasets presented in this study can be found in online repositories. The names of the repository/repositories and accession number(s) can be found at: https://www.ncbi.nlm.nih.gov/, SRA accessions: SRR11960652-SRR11960732.

## Ethics Statement

The animal study was reviewed and approved by The University of Queensland AnimalEthicsSVS/487/15/KIBBLE. Written informed consent was obtained from the owners for the participation of their animals in this study.

## Author Contributions

HR, JG, EM, RS, and SB were involved in the study design and analyses. HR, BF, and SA were involved in the sample processing and bioinformatics analyses. All authors contributed significantly to the work, read, and approved the final version of the manuscript which is not being considered for publication elsewhere.

## Conflict of Interest

The authors declare that the research was conducted in the absence of any commercial or financial relationships that could be construed as a potential conflict of interest.

## Footnotes

^1^PubMLST. This publication made use of the PubMLST website (https://pubmlst.org/) developed by Keith Jolley (Jolley et al. (36) Wellcome Open Res 2018 3:124 [version 1; referees: 2 approved]) and sited at the University of Oxford. The development of that website was funded by the Wellcome Trust.

## References

[1] Ruscher C Lübke-Becker A Wleklinski C.-G Soba A Wieler LH Walther B. Prevalence of methicillin-resistant *Staphylococcus pseudintermedius* isolated from clinical samples of companion animals and equidaes. Vet Microbiol. (2009) 136:197–201. 10.1016/j.vetmic.2008.10.023 19097710

[2] Somayaji R Priyantha MA Rubin JE Church D. Human infections due to *Staphylococcus pseudintermedius*, an emerging zoonosis of canine origin: report of 24 cases. Diagn Micr Infect Dis. (2016) 85:471–6. 10.1016/j.diagmicrobio.2016.05.008 27241371

[3] Cohn LA Middleton JR. A veterinary perspective on methicillin-resistant staphylococci. J Vet Emerg Crit Care. (2010) 20:31–45. 10.1111/j.1476-4431.2009.00497.x 20230433

[4] Zhang K McClure J.-A. Elsayed S Conly JM. Novel Staphylococcal cassette chromosome mec type, tentatively designated type VIII. Harboring class A mec and type 4 ccr gene complexes in a canadian epidemic strain of methicillin-resistant *Staphylococcus aureus*. Antimicrob Agents Chemother. (2009) 53:531–40. 10.1128/AAC.01118-08 19064897 PMC2630601

[5] Moodley A Damborg P Nielsen SS. Antimicrobial resistance in methicillin susceptible and methicillin resistant *Staphylococcus pseudintermedius* of canine origin: Literature review from 1980 to 2013. Vet Microbiol. (2014) 171:337–41. 10.1016/j.vetmic.2014.02.008 24613081

[6] Worthing KA Abraham S Coombs GW Pang S Saputra S Jordan D. Clonal diversity and geographic distribution of methicillin-resistant *Staphylococcus pseudintermedius* from Australian animals: discovery of novel sequence types. Vet. Microbiol. (2018) 213:58–65. 10.1016/j.vetmic.2017.11.018 29292005

[7] Worthing KA Brown J Gerber L Trott DJ Abraham S Norris JM. Methicillin-resistant staphylococci amongst veterinary personnel, personnel-owned pets, patients and the hospital environment of two small animal veterinary hospitals. Vet Microbiol. (2018) 223:79–85. 10.1016/j.vetmic.2018.07.021 30173756

[8] Saputra S Jordan D Worthing KA Norris JM Wong HS Abraham R. Antimicrobial resistance in coagulase-positive staphylococci isolated from companion animals in Australia: a one year study. PLoS ONE. (2017) 12:e0176379. 10.1371/journal.pone.0176379 28430811 PMC5400250

[9] Worthing KA Abraham S Pang S Coombs GW Saputra S Jordan D. Molecular characterization of methicillin-resistant *Staphylococcus aureus* isolated from Australian animals and veterinarians. Microb Drug Resist. (2018) 24:203–12. 10.1089/mdr.2017.0032 28598251

[10] Pires dos Santos T Damborg P Moodley A Guardabassi L. Systematic review on global epidemiology of methicillin-resistant *Staphylococcus pseudintermedius*: inference of population structure from multilocus sequence typing data. Front Microbiol. (2016) 7:1599. 10.3389/fmicb.2016.01599 27803691 PMC5067483

[11] Börjesson S Gómez-Sanz E Ekström K Torres C Grönlund U. *Staphylococcus pseudintermedius* can be misdiagnosed as *Staphylococcus aureus* in humans with dog bite wounds. *Eur. J Clin*. Microbiol. Infect. Dis. (2015) 34:839–844. 10.1007/s10096-014-2300-y 25532507

[12] Stegmann R Burnens A Maranta CA Perreten V. Human infection associated with methicillin-resistant *Staphylococcus pseudintermedius* ST71. J Antimicrob Chemother. (2010) 65:2047–8. 10.1093/jac/dkq241 20601356

[13] Aires-de-Sousa M. Methicillin-resistant *Staphylococcus aureus* among animals: current overview. Clin Microbiol Infect. (2017) 23:373–80. 10.1016/j.cmi.2016.11.002 27851997

[14] Balachandran M Bemis DA Kania SA. Expression and function of protein A in *Staphylococcus pseudintermedius*. Virulence. (2017) 9:390–401. 10.1080/21505594.2017.1403710 29157101 PMC5955199

[15] Dziewanowska K Edwards VM Deringer JR Bohach GA Guerra DJ. Comparison of the β-toxins from *Staphylococcus aureus* and *Staphylococcus intermedius*. Arch Biochem Biophys. (1996) 335:102–8. 10.1006/abbi.1996.0486 8914839

[16] Etienne J. Panton-valentine leukocidin: a marker of severity for *Staphylococcus aureus* infection? Clin Infect Dis. (2005) 41:591–3. 10.1086/432481 16080078

[17] Garbacz K Zarnowska S Piechowicz L Haras K. Pathogenicity potential of *Staphylococcus pseudintermedius* strains isolated from canine carriers and from dogs with infection signs. Virulence. (2013) 4:255–9. 10.4161/viru.23526 23328490 PMC3711984

[18] Asanin J Misic D Aksentijevic K Tambur Z Rakonjac B Kovacevic I. Genetic profiling and comparison of human and animal methicillin-resistant *Staphylococcus aureus* (MRSA) isolates from Serbia. Antibiotics. 8:26. 10.3390/antibiotics8010026 30884836 PMC6466565

[19] Feßler AT Schuenemann R Kadlec K Hensel V Brombach J Murugaiyan J. Methicillin-resistant *Staphylococcus aureus* (MRSA) and methicillin-resistant *Staphylococcus pseudintermedius* (MRSP) among employees and in the environment of a small animal hospital. Vet Microbiol. (2018) 221:153–8. 10.1016/j.vetmic.2018.06.001 29981702

[20] Han J.-I. Rhim H Yang C.-H. Park M.. Molecular characteristics of new clonal complexes of *Staphylococcus pseudintermedius* from clinically normal dogs. Vet Q. (2018) 38:14–20. 10.1080/01652176.2017.1400710 29135361 PMC6830971

[21] Perreten V Kadlec K Schwarz S Gronlund Andersson U Finn M Greko C. Clonal spread of methicillin-resistant *Staphylococcus pseudintermedius* in Europe and North America: an international multicentre study. J Antimicrob Chemother. (2010) 65:1145–54. 10.1093/jac/dkq078 20348087

[22] Wang J Liu Y Wan D Fang X Li T Guo Y. Whole-genome sequence of *Staphylococcus aureus* strain LCT-SA112. J. Bacteriol. (2012) 194:4124. 10.1128/JB.00710-12 22815443 PMC3416511

[23] Steen S. Meticillin-resistant strains of *Staphylococcus pseudintermedius* in companion animals. Vet Rec. (2011) 169:53. 10.1136/vr.d4247 21742696

[24] Smith J Amador S McGonagle CJ Needle D Gibson R Andam CP. Population genomics of *Staphylococcus pseudintermedius* in companion animals in the United States. Coomun Biol. (2020) 3:282. 10.1038/s42003-020-1009-y 32503984 PMC7275049

[25] Feng Y Tian W Lin D Luo Q Zhou Y Yang T. Prevalence and characterization of methicillin-resistant *Staphylococcus pseudintermedius* in pets from South China. Vet Microbiol. (2012) 160:517–24. 10.1016/j.vetmic.2012.06.015 22770517

[26] Kjellman EE Slettemeas JS Small H Sunde M. Methicillin-resistant *Staphylococcus pseudintermedius* (MRSP) from healthy dogs in Norway - occurrence, genotypes and comparison to clinical MRSP. MicrobiologyOpen. (2015) 4:857–66. 10.1002/mbo3.258 26423808 PMC4694142

[27] Lee GY Yang SJ. Comparative assessment of genotypic and phenotypic correlates of *Staphylococcus pseudintermedius* strains isolated from dogs with otitis externa and healthy dogs. Comp Immunol Microbiol Infect Dis. (2019) 70:101376. 10.1016/j.cimid.2019.101376 31703937

[28] Clinical and Laboratory Standards Institute. Performance standards for antimicrobial disk and dilution susceptibility tests for bacteria isolated from animals. In: CLSI Standard VET08. 4th ed. Wayne, PA: ClinicaI and Laboratory Standards Institute (2018)

[29] Clinical and Laboratory Standards Institute. Performance Standards for Antimocrobial Susceptibility Testing; Twenty-Second Informational Supplement. CLSI Supplement M100. 30th edn. Wayne, PA: ClinicaI and Laboratory Standards Institute (2020).

[30] Sweeney MT Lubbers BV Schwarz S Watts JL. Applying definitions for multidrug resistance, extensive drug resistance and pandrug resistance to clinically significant livestock and companion animal bacterial pathogens. J Antimicrob Chemother. (2018) 73:1460–3. 10.1093/jac/dky043 29481657

[31] Geha DJ Uhl JR Gustaferro CA Persing DH. Multiplex PCR for the identification of methicillin resistant Staphylococci in the clinical laboratory. J Clin Microbiol. (1994) 32:1768–72. 10.1128/JCM.32.7.1768-1772.1994 7929772 PMC263789

[32] Sasaki T Tsubakishita S Tanaka Y Sakusabe A Ohtsuka M Hirotaki S. Multiplex-PCR method for species identification of coagulase-positive staphylococci. J Clin. Microbiol. (2010) 48:765–9. 10.1128/JCM.01232-09 20053855 PMC2832457

[33] Bolger AM Lohse M Usadel B. Trimmomatic: a flexible trimmer for illumina sequence data. Bioinformatics. (2014) 30:2114–20. 10.1093/bioinformatics/btu170 24695404 PMC4103590

[34] Bankevich A Nurk S Antipov D Gurevich AA Dvorkin M Kulikov AS. SPAdes: A new genome assembly algorithm and its applications to single-cell sequencing. J Comput Biol. (2012) 19:455–77. 10.1089/cmb.2012.0021 22506599 PMC3342519

[35] Inouye M Dashnow H Raven LA Schultz MB Pope BJ. SRST2: Rapid genomic surveillance for public health and hospital microbiology labs. Genome Med. (2014) 6:90. 10.1186/s13073-014-0090-6 25422674 PMC4237778

[36] Jolley KA Bray JE Maiden MCJ. Open-access bacterial population genomics: BIGSdb software, the PubMLST.org website and their applications. Wellcome Open Res. (2018) 3:124. 10.12688/wellcomeopenres.14826.1 30345391 PMC6192448

[37] Kaya H Hasman H Larsen J Stegger M Johannesen TB Allesøe RL. SCCmecFinder, a web-based tool for typing of staphylococcal cassette chromosome mec in *Staphylococcus aureus* using whole-genome sequence data. mSphere. (2018) 3:e00612–7. 10.1128/mSphere.00612-17 29468193 PMC5812897

[38] Feldgarden M Brover V Haft DH Prasad AB Slotta DJ Tolstoy I. Validating the AMRFinder tool and resistance gene database by using antimicrobial resistance genotype-phenotype correlations in a collection of isolates. Antimicrob Agents Chemother. (2019) 63:e00483–e00419. 10.1128/AAC.00483-19 31427293 PMC6811410

[39] Chen L. VFDB: a reference database for bacterial virulence factors. Nucleic Acids Res. (2004) 33:D325–8. 10.1093/nar/gki008 15608208 PMC539962

[40] Carver TJ Rutherford KM Berriman M Rajandream MA Barrell BG Parkhill J. ACT: the Artemis comparison tool. Bioinformatics. (2005) 21:3422–23. 10.1093/bioinformatics/bti553 15976072

[41] Treangen TJ Ondov BD Koren S Phillippy AM. The Harvest suite for rapid core-genome alignment and visualization of thousands of intraspecific microbial genomes. Genome Biol. (2014) 15:524. 10.1101/007351 25410596 PMC4262987

[42] Croucher NJ Page AJ Connor TR Delaney AJ Keane JA Bentley SD. Rapid phylogenetic analysis of large samples of recombinant bacterial whole genome sequences using Gubbins. Nucleic Acids Res. (2015) 43:e15. 10.1093/nar/gku1196 25414349 PMC4330336

[43] Stamatakis A. RAxML version 8: a tool for phylogenetic analysis and post-analysis of large phylogenies. Bioinformatics. (2014) 30:1312–3. 10.1093/bioinformatics/btu033 24451623 PMC3998144

[44] Rambaut A. FigTree. (2018). Available online at: http://tree.bio.ed.ac.uk/software/figtree/ (accessed July 01, 2020).

[45] He Z Zhang H Gao S Lercher MJ Chen WH Hu S. Evolview v2: an online visualization and management tool for customized and annotated phylogenetic trees. Nucleic Acids Res. (2016) 44:W236–41. 10.1093/nar/gkw370 27131786 PMC4987921

[46] Australian Government. Importance Ratings and Summary of Antibacterial Uses in Human and Animal Health in Australia. Canberra (2018).

[47] Jafari M Ansari-Pour N. Why, when and how to adjust your P values? Cell J. (2019) 20:604–7. 10.22074/cellj.2019.5992 30124010 PMC6099145

[48] Clinical and Laboratory Standards Institute. Performance Standards for Antimocrobial Susceptibility Testing. CLSI Supplement M100. 29th edn. Wayne, PA: ClinicaI and Laboratory Standards Institute (2019).

[49] Beck KM Waisglass SE Dick HLN Weese JS. Prevalence of meticillin-resistant *Staphylococcus pseudintermedius* (MRSP) from skin and carriage sites of dogs after treatment of their meticillin-resistant or meticillin-sensitive staphylococcal pyoderma. Vet Dermatol. (2012) 23:369–75. 10.1111/j.1365-3164.2012.01035.x 22364707

[50] Gómez-Sanz E Torres C Lozano C Zarazaga M. High diversity of *Staphylococcus aureus* and *Staphylococcus pseudintermedius* lineages and toxigenic traits in healthy pet-owning household members. Underestimating normal household contact? Comp Immunol Microb. (2013) 36:83–94. 10.1016/j.cimid.2012.10.001 23153600

[51] Gronthal T Ollilainen M Eklund M Piiparinen H Gindonis V Junnila J. Epidemiology of methicillin resistant *Staphylococcus pseudintermedius* in guide dogs in Finland. Acta Vet Scand. (2015) 57:37. 10.1186/s13028-015-0129-8 26183814 PMC4504442

[52] Tabatabaei S Najafifar A Askari Badouei M Zahraei Salehi T Ashrafi Tamai I Khaksar E. Genetic characterisation of methicillin-resistant *Staphylococcus aureus* and *Staphylococcus pseudintermedius* in pets and veterinary personnel in Iran: new insights into emerging methicillin-resistant *S. pseudintermedius* (MRSP). J Glob Antimicrob Re. (2019) 16:6–10. 10.1016/j.jgar.2018.08.022 30172831

[53] Abraham JL Morris DO Griffeth GC Shofer FS Rankin SC. Surveillance of healthy cats and cats with inflammatory skin disease for colonization of the skin by methicillin-resistant coagulase-positive staphylococci and *Staphylococcus schleiferi* ssp. schleiferi. Vet. Dermatol. (2007) 18:252–9. 10.1111/j.1365-3164.2007.00604.x 17610491

[54] Lilenbaum W Esteves AL Souza GN. Prevalence and antimicrobial susceptibility of staphylococci isolated from saliva of clinically normal cats. Lett Appl Microbiol. (1999) 28:448–52. 10.1046/j.1365-2672.1999.00540.x 10389262

[55] Couto N Pomba C Moodley A Guardabassi L. Prevalence of methicillin-resistant staphylococci among dogs and cats at a veterinary teaching hospital in Portugal. Vet Rec. (2011) 169:72. 10.1136/vr.c6948 21502197

[56] Hanselman BA Kruth S Weese JS. Methicillin-resistant staphylococcal colonization in dogs entering a veterinary teaching hospital. Vet Microbiol. (2008) 126:277–81. 10.1016/j.vetmic.2007.06.015 17643874

[57] Loeffler A Boag AK Sung J Lindsay JA Guardabassi L Dalsgaard A. Prevalence of methicillin-resistant *Staphylococcus aureus* among staff and pets in a small animal referral hospital in the UK. J Antimicrob Chemother. (2005) 56:692–7. 10.1093/jac/dki312 16141276

[58] Loeffler A Pfeiffer DU Lindsay JA Soares Magalhaes RJ Lloyd DH. Prevalence of and risk factors for MRSA carriage in companion animals: a survey of dogs, cats and horses. Epidemiol Infect. (2011) 139:1019–28. 10.1017/S095026881000227X 20943000

[59] Weese JS van Duijkeren E. Methicillin-resistant *Staphylococcus aureus* and *Staphylococcus pseudintermedius* in veterinary medicine. Vet Microbiol. (2010) 140:418–29. 10.1016/j.vetmic.2009.01.039 19246166

[60] Bergot M Martins-Simoes P Kilian H Châtre P Worthing KA Norris JM. Evolution of the population structure of *Staphylococcus pseudintermedius* in France. Front Microbiol. (2018) 9:3055. 10.3389/fmicb.2018.03055 30619143 PMC6300469

[61] Nisa S Bercker C Midwinter AC Bruce I Graham CF Venter P. Combining MALDI-TOF and genomics in the study of methicillin resistant and multidrug resistant *Staphylococcus pseudintermedius* in New Zealand. Sci Rep. (2019) 9:1217. 10.1038/s41598-018-37503-9 30718644 PMC6361924

[62] Kizerwetter-Swida M Chrobak-Chmiel D Rzewuska M Binek M. Changes in the population structure of canine methicillin-resistant *Staphylococcus pseudintermedius* in Poland. Vet Microbiol. (2017) 208:106–9. 10.1016/j.vetmic.2017.07.025 28888624

[63] Australian Government Department of Agriculture Water and Environment. Export. (2020). Available online at: https://www.agriculture.gov.au/export (accessed May 8, 2020).

[64] Australian Government Department of Agriculture Water and Environment. Import. (2020). Available online at: https://www.agriculture.gov.au/import (accessed May 8, 2020).

[65] International Working Group on the Classification of Staphylococcal Cassette Chromosome Elements (IWG-SCC). Classification of staphylococcal cassette chromosome mec (SCCmec): guidelines for reporting novel SCCmec elements. Antimicrob Agents Chemother. (2009) 53:4961–7. 10.1128/AAC.00579-09 19721075 PMC2786320

[66] Martins Simões P Rasigade JP Lemriss H Butin M Ginevra C Lemriss S. Characterization of a novel composite Staphylococcal cassette chromosome mec (SCCmec-SCCcad/ars/cop) in the neonatal sepsis-associated *Staphylococcus capitis* pulsotype NRCS-A. Antimicrob Agents Chemother. (2013) 57:6354. 10.1128/AAC.01576-13 24060879 PMC3837888

[67] Abouelkhair MA Bemis DA Giannone RJ Frank LA Kania SA. Characterization of a leukocidin identified in *Staphylococcus pseudintermedius*. PLoS ONE. (2018) 13:e0204450. 10.1371/journal.pone.0204450 30261001 PMC6160070

[68] Bannoehr J Guardabassi L. *Staphylococcus pseudintermedius* in the dog: taxonomy, diagnostics, ecology, epidemiology and pathogenicity. Vet Dermatol. (2012) 23:253–66. 10.1111/j.1365-3164.2012.01046.x 22515504

[69] Ben Zakour N Beatson S van den Broek A Thoday K Fitzgerald R. Comparative genomics of the *Staphylococcus intermedius* group of animal pathogens. Front Cell Infect Mi. (2012) 2:44. 10.3389/fcimb.2012.00044 22919635 PMC3417630

[70] Frees D Qazi SNA Hill PJ Ingmer H. Alternative roles of ClpX and ClpP in *Staphylococcus aureus* stress tolerance and virulence. Mol Microbiol. (2003) 48:1565–78. 10.1046/j.1365-2958.2003.03524.x 12791139

[71] Sakarya S Ertugrul MB Öztürk T Gökbulut C. Effect of pharynx epithelial cells surface desialylation on receptor-mediated adherence of *Staphylococcus aureus*. J Appl Microbiol. (2010) 108:1313–22. 10.1111/j.1365-2672.2009.04525.x 19778351

[72] Banovic F Linder K Olivry T. Clinical, microscopic and microbial characterization of exfoliative superficial pyoderma-associated epidermal collarettes in dogs. Vet Dermatol. (2017) 28:e107–23. 10.1002/9781119278368.ch5.3 27426474

[73] Zukancic A Khan MA Gurmen SJ Gliniecki QM Moritz-Kinkade DL Maddox CW. Staphylococcal Protein A (spa) locus is a hot spot for recombination and horizontal gene transfer in Staphylococcus pseudintermedius. mSphere. (2020) 5:e00666–20. 10.1128/mSphere.00666-20 33115833 PMC7593597

[74] AnandaChitra M Jayanthy C Nagarajan B. Virulence genes detection and antimicrobial susceptibility of *Staphylococcus pseudintermedius* isolates from canine skin infection in Chennai, India. Proc Natl Acad Sci India Section B: Biol Sci. (2018) 88:355–61. 10.1007/s40011-016-0760-9

