# Supplementary material for: Molecular Epidemiology of Clinical and Colonizing Methicillin-Resistant Staphylococcus Isolates in Companion Animals
Source: Front Vet Sci. 2021 Apr 23;8:620491. doi: 10.3389/fvets.2021.620491 (PMC8102687; doi:10.3389/fvets.2021.620491)
Supplement: Supplementary file 1 [file Data_Sheet_1.docx]

**Supplementary files**

**Table 1. The percentage distribution of methicillin resistant *Staphylococcus pseudintermedius* clones by attribute data.**

| Clones | 496 | 749 |
| --- | --- | --- |
| Species | | |
| Dog | 75 | 100 |
| Cat | 28 | 0 |
| Female | 31 | 67 |
| Male | 72 | 33 |
| Age category | | |
| ≤ 1 year | 31 | 11 |
| 1 to 4 years | 19 | 33 |
| 4 to 7 years | 11 | 11 |
| 7 to 10 years | 28 | 11 |
| ≥ 10 years | 11 | 33 |
| Neuter Status | | |
| Neutered | 47 | 67 |
| Entire | 33 | 33 |
| Sampling Location |  |  |
| Clinic A | 8 | 56 |
| Clinic B | 22 | 33 |
| Clinic C | 39 | 11 |
| Shelter | 47 | 0 |
| Antibiotic use in year prior to sampling* | | |
| Yes | 78 | 56 |
| No | 22 | 44 |
| Glucocorticoid use in year prior to sampling | | |
| Yes | 19 | 11 |
| No | 81 | 89 |
| Consultation type |  |  |
| General Practice | 6 | 22 |
| Internal Medicine | 31 | 56 |
| Dermatology | 17 | 11 |
| Surgery | 0 | 11 |
| Shelter animals | 47 | 0 |

**Supplementary Table 2. The resistence and virulence gene profile categories used for Fisher’s Exacts tests.**

| Categories used in analysis | |
| --- | --- |
| Resistance Genes | |
| 1 | Contained genes encoding resistance to 1^st^ line (aminoglycosides, beta lactams, tetracyclines, chloramphenicol), 2^nd^ line (aminoglycosides, beta lactams, sulphonamides, macrolides and lincosamides) , 3^rd^ line (fluoroquinolones) and important human antimicrobials (aminoglycosides: amikacin (*aph-III*)) |
| 2 | Contained genes encoding resistance to 1^st^ line, 2^nd^ line and important human antimicrobials |
| 3 | Contained genes encoding resistance to 1^st^ line and 2^nd^ line antimicrobials |
|  | **Virulence Genes** |
| 1 (most common virulence gene profiles) | *LukF-P, SrrA, clpX, coa, ebpS, hrtA, lip, sarA, sarR, sarZ, siet, speta, spsA, spsB, spsC, spsE, spsG, spsH, spsK, spsM, spsN, spsR, hlgB, clpP* |
|  | *LukF-P, SrrA, clpX, coa, ebpS, hrtA, lip, sarA, sarR, sarZ, siet, speta, spsA, spsB, spsC, spsE, spsG, spsH, spsK, spsM, spsN, spsP, spsQ, spsR, hlgB, clpP* |
|  |  |
| 2 | ***LukF-P, SrrA, clpX, coa, ebpS, hrtA, lip, sarA, sarR, sarZ, siet, speta, spsB, spsR, hlgB, clpP****, spsA, spsC, spsE, spsG, spsH, spsM, spsN, spsP, spsQ* |
|  | ***LukF-P, SrrA, clpX, coa, ebpS, hrtA, lip, sarA, sarR, sarZ, siet, speta, spsB, spsR, hlgB, clpP****, spsA, spsC, spsE, spsG, spsH, spsK, spsN* |
|  | ***LukF-P, SrrA, clpX, coa, ebpS, hrtA, lip, sarA, sarR, sarZ, siet, speta, spsB, spsR, hlgB, clpP****, spsA, spsE, spsG, spsH, spsK, spsM, spsN* |
|  | ***LukF-P, SrrA, clpX, coa, ebpS, hrtA, lip, sarA, sarR, sarZ, siet, speta, spsB, spsR, hlgB, clpP****, spsC, spsE, spsG, spsH, spsK, spsM, spsN* |
|  | ***LukF-P, SrrA, clpX, coa, ebpS, hrtA, lip, sarA, sarR, sarZ, siet, speta, spsB, spsR, hlgB, clpP****, spsA, spsC, spsE, spsG, spsK, spsN* |
|  | ***LukF-P, SrrA, clpX, coa, ebpS, hrtA, lip, sarA, sarR, sarZ, siet, speta, spsB, spsR, hlgB, clpP****, spsC* |
|  |  |
| 3 | ***LukF-P, SrrA, clpX, coa, ebpS, hrtA, lip, nanB, sarA, sarR, sarZ, siet, speta, spsA, spsB, spsE, spsK, spsM, spsN, spsR, hlgB, clpP****, spsC, spsG, spsH* |
|  | ***LukF-P, SrrA, clpX, coa, ebpS, hrtA, lip, nanB, sarA, sarR, sarZ, siet, speta, spsA, spsB, spsE, spsK, spsM, spsN, spsR, hlgB, clpP****, spsC, spsG, spsH, spsI, spsP, spsQ* |
|  | ***LukF-P, SrrA, clpX, coa, ebpS, hrtA, lip, nanB, sarA, sarR, sarZ, siet, speta, spsA, spsB, spsE, spsK, spsM, spsN, spsR, hlgB, clpP****, spsI* |
|  |  |
| 4 | ***LukF-P, SrrA, clpX, coa, sarA, spsK, hlgB, clpP****, ebpS, hrtA, sarR, sarZ, siet, speta, spsA, spsB, spsC, spsE, spsG, spsH, spsM, spsN,spsQ, spsR* |
|  | ***LukF-P, SrrA, clpX, coa, sarA, spsK, hlgB, clpP****, ebpS, hrtA, lip, sarR, sarZ, speta, spsA, spsB, spsE, spsH, spsN, spsR* |
|  | ***LukF-P, SrrA, clpX, coa, sarA, spsK, hlgB, clpP****, ebpS, hrtA, lip, sarR, sarZ, siet, spsA, spsB, spsC, spsG, spsH, spsN, spsR* |
|  | ***LukF-P, SrrA, clpX, coa, sarA, spsK, hlgB, clpP****, ebpS, sarR, siet, speta, spsB, spsE, spsG, spsR* |
|  | ***LukF-P, SrrA, clpX, coa, sarA, spsK, hlgB, clpP****, lip, sarR, sarZ, spsA, spsB, spsE, spsH, spsR* |
|  | ***LukF-P, SrrA, clpX, coa, sarA, spsK, hlgB, clpP****, hrtA, sarZ, speta, spsA, spsH, spsR* |
|  | ***LukF-P, SrrA, clpX, coa, sarA, spsK, hlgB, clpP****, hrtA, sarR, sarZ, siet, speta, spsA, spsB, spsH, spsQ* |
|  |  |
| 5 | ***LukF-P, SrrA, clpX, nanB, sarA, sarR, siet, clpP****, coa, ebpS, hrtA, lip, sarZ, speta, spsA, spsB, spsC, spsE, spsG, spsH, spsK, spsM, spsN, spsR* |
|  | ***LukF-P, SrrA, clpX, nanB, sarA, sarR, siet, clpP****, coa, ebpS, hrtA, lip, sarZ, speta, spsA, spsC, spsE, spsG, spsI, spsM, spsR, hlgB* |
|  | ***LukF-P, SrrA, clpX, nanB, sarA, sarR, siet, clpP****, coa, hrtA, lip, sarZ, speta, spsA, spsB, spsC, spsE, spsH, spsI, spsK, spsN, hlgB* |
|  | ***LukF-P, SrrA, clpX, nanB, sarA, sarR, siet, clpP****, coa, hrtA, sarZ, speta, spsA, spsE, spsH, spsK, spsM, spsR, hlgB* |
|  | ***LukF-P, SrrA, clpX, nanB, sarA, sarR, siet, clpP****, coa, sarZ, spsB, spsE, spsK, spsN, spsR, hlgB* |
|  | ***LukF-P, SrrA, clpX, nanB, sarA, sarR, siet, clpP****, ebpS, hrtA, lip, sarZ, speta, spsA, spsB, spsE, spsH, spsK, spsN, spsR, hlgB* |
|  | ***LukF-P, SrrA, clpX, nanB, sarA, sarR, siet, clpP****, ebpS, hrtA, sarZ, spsA, spsB, spsH, spsK, spsN,spsQ, spsR, hlgB* |
|  | ***LukF-P, SrrA, clpX, nanB, sarA, sarR, siet, clpP****, ebpS, hrtA, lip, spsB, spsH, spsK, spsR, hlgB* |
|  |  |
| 6 | ***SrrA, ebpS, hrtA, sarA, sarR, sarZ, speta, hlgB, clpP****, LukF-P, clpX, siet, spsB, spsC, spsE, spsK, spsN* |
|  | ***SrrA, ebpS, hrtA, sarA, sarR, sarZ, speta, hlgB, clpP****, LukF-P, clpX, lip, siet, spsB, spsK, spsN, spsR* |
|  | ***SrrA, ebpS, hrtA, sarA, sarR, sarZ, speta, hlgB, clpP****, LukF-P, coa, lip, spsB, spsE, spsH, spsK, spsR* |
|  | ***SrrA, ebpS, hrtA, sarA, sarR, sarZ, speta, hlgB, clpP****, LukF-P, coa, siet, spsA, spsB, spsK, spsN, spsP* |
|  | ***SrrA, ebpS, hrtA, sarA, sarR, sarZ, speta, hlgB, clpP****, coa, lip, spsA, spsB, spsC, spsE, spsG, spsK, spsM, spsN, spsR* |
|  | ***SrrA, ebpS, hrtA, sarA, sarR, sarZ, speta, hlgB, clpP****, clpX, coa, lip, siet, spsA, spsC, spsE, spsG, spsH, spsM, spsN,spsP, spsQ, spsR* |

Shared gene combinations in the virulence gene categories are bolded. The functions of the genes are as follows: toxins [*LukF-P, hlgB* (gamma haemolysin component B)], exfoliative toxin [*siet* (*S. intermedius* gene for exfoliative toxin), *speta* (exfoliative toxin A)], accessory gene regulators [s*rrA* (staph respiratory response protein), *sarA* (staphylococcal accessory regulator A)*, sarR* (transcriptional regulator)*, sarZ* (transcriptional regulator)], cell wall anchored proteins [*ebpS* (elastin-binding protein), *spsC, spsE*, *spsH* , *spsK, spsA, spsB, spsG, spsI, spsM, spsN, spsP, spsQ, and spsR* (*S. pseudintermedius* surface proteins)], exoenzymes [*nanB* (putative sialidase toxin), *coa* (staphylocoagulase)*, hrtA* (heme efflux system ATPase HrtA) *,lip* (triacylglycerol lipase)], *clpX* [ATP-dependent protease ATP-binding subunit ClpX], and a protease *clpP* (Abouelkhair et al., 2018; Bannoehr et al., 2012; Ben Zakour et al., 2012; Bergot et al., 2018; Frees et al., 2003).

**Supplementary Table 3. The number of animals sampled per clinic and methicillin resistant *Staphylococcus aureus* (MRSA) and methicillin resistant *Staphylococcus pseudintermedius* (MRSP) isolated for each.**

| Location sampled | Number sampled | Number of carriers (%) | |
| --- | --- | --- | --- |
| Clinics | | **MRSA** | **MRSP** |
| Clinic A total | **291** |  |  |
| Dogs | 255 | 0 | 11 (4%) |
| Cats | 36 | 0 | 0 |
| Clinic B total | **119** |  |  |
| Dogs | 102 | 0 | 14 (14%) |
| Cats | 17 | 0 | 0 |
| Clinic C total | **34** |  |  |
| Dogs | 30 | 0 | 9 (30%) |
| Cats | 4 | 0 | 0 |
| Clinic D total | **23** |  |  |
| Dogs | 22 | 0 | 0 |
| Cats | 1 | 0 | 0 |
| Clinic F total | **23** |  |  |
| Cats | 23 | 0 | 0 |
| Total clinic Dogs | **409** | **0** | **34 (8%)** |
| Total clinic Cats | **81** | **0** | **0** |
| Shelter animals | | | |
| Shelter A total | **105** |  |  |
| Dogs | 19 | 0 | 7 (37%) |
| Cats | 86 | 0 | 9 (10%) |
| Shelter B total |  |  |  |
| Dogs | 30 | 0 | 0 |
| Cats | 41 | 0 | 0 |
| Shelter C total | **12** |  |  |
| Dogs | 12 | 0 | 0 |
| Total shelter Dogs | **61** | **0** | **7 (11%)** |
| Total shelter Cats | **127** | **0** | **9 (7%)** |
| Total animals sampled in study | | | |
| Total Dogs sampled | **470** | **0** | **41 (9%)** |
| Total Cats sampled | **208** | **0** | **9 (4%)** |

**Supplementary Figure 1. A global phylogenetic tree including isolates from this study and around the world.**
