# Supplementary figures and images for: Molecular Epidemiology of Clinical and Colonizing Methicillin-Resistant Staphylococcus Isolates in Companion Animals
Source: Front Vet Sci. 2021 Apr 23;8:620491. doi: 10.3389/fvets.2021.620491 (PMC8102687; doi:10.3389/fvets.2021.620491)

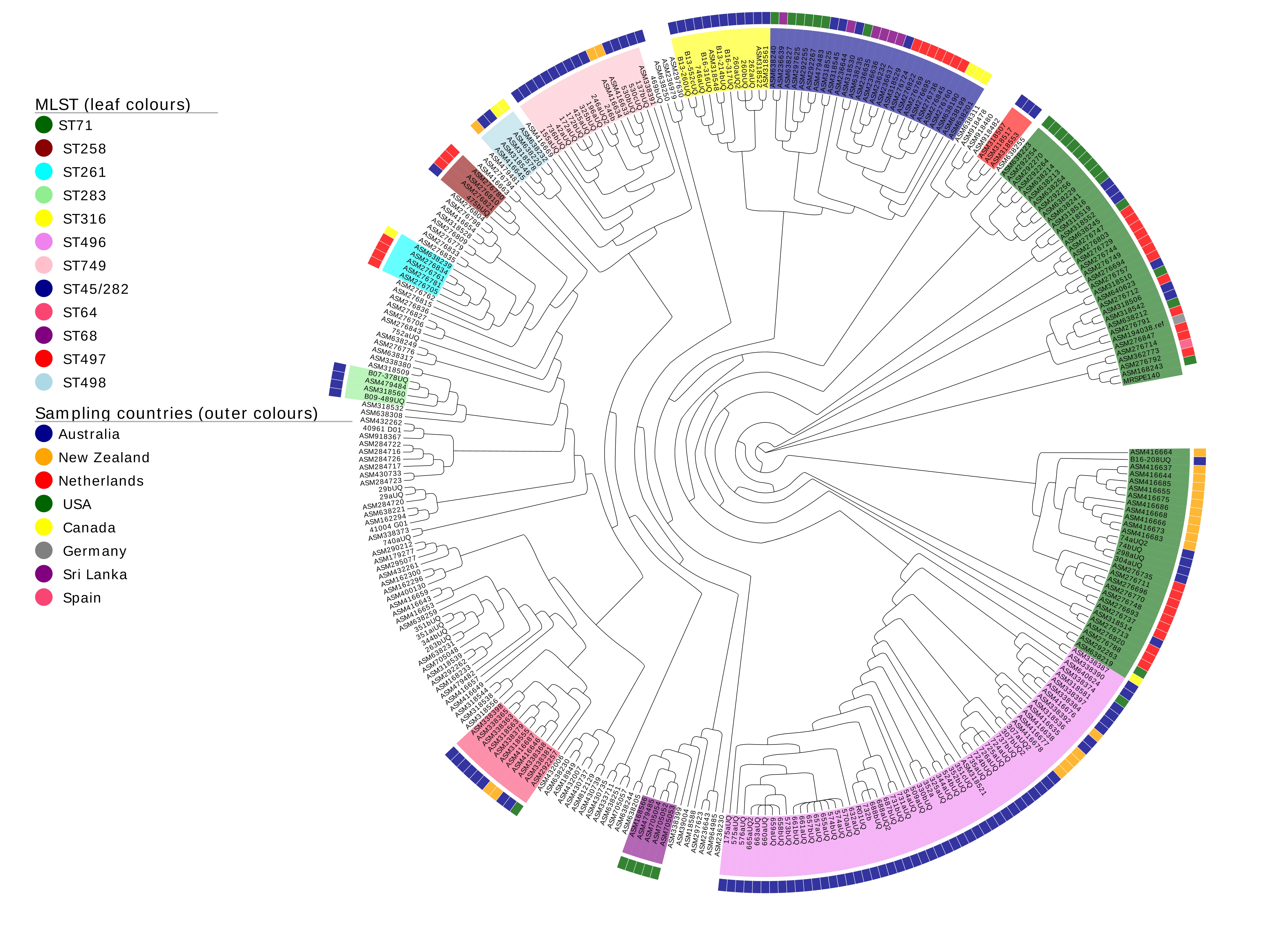

Supplement: Supplementary file 2 [file Image_1.JPEG]
